# Exploring nutrient limitation for polyhydroxyalkanoates synthesis by newly isolated strains of *Aeromonas* sp. using biodiesel-derived glycerol as a substrate

**DOI:** 10.7717/peerj.5838

**Published:** 2018-10-23

**Authors:** Justyna Możejko-Ciesielska, Tomasz Pokoj

**Affiliations:** 1 Department of Microbiology and Mycology, Faculty of Biology and Biotechnology, University of Warmia and Mazury in Olsztyn, Olsztyn, Poland; 2 Department of Environmental Biotechnology, Faculty of Environmental Sciences, University of Warmia and Mazury in Olsztyn, Olsztyn, Poland

**Keywords:** *Aeromonas* sp, Polyhydroxyalkanoates, Biodiesel-derived glycerol, P(3HB), P(3HB-*co*-HV)

## Abstract

*Aeromonas* spp. strains isolated from activated sludge in a municipal wastewater treatment plant were found to be able to synthesize polyhydroxyalkanoates (PHA) utilizing pure and crude glycerol. The 16S rRNA gene sequence of the isolates exhibited similarity to *Aeromonas hydrophila*, *A. aquatica*, and *A. salmonicida*. Our results confirmed that the adequate supply of nitrogen and phosphorus during culture in 250-ml shake flasks did not stimulate the synthesis of PHAs. The results indicate that the PHA content of cells was higher under a phosphorus-limiting environment compared to nitrogen starvation. In the two-stage cultivation using glucose (in the first step) and crude glycerol from biodiesel industry (in the second step) as a component of the growth medium, the analyzed strains grew to 3.06 g/l of cell dry weight containing up to 22% of PHAs. Furthermore, during the same culture strategy up to 42% of PHAs were extracted, when in the second step of the process, *Aeromonas* sp. AC_03 was grown on pure glycerol under phosphorus limitation. The purified biopolymer was confirmed to be polyhydroxybutyrate. *Aeromonas* sp. AC_02 was also capable to accumulate the poly(3-hydroxybutyrate-*co*-3-hydroxyvalerate) copolymer when pure glycerol was added as a substrate under nitrogen-deficiency one-step bioprocess. Our results confirm that due to the biopolymer productivity, newly isolated strains could be exploited for obtaining valuable biopolymers using wastes generated from biodiesel industry.

## Introduction

With the exhaustion of fossil fuels and increasing environmental issues, an alternative to conventional petrochemical plastics has attracted much attention in recent years. Microorganisms can be used to synthesize a number of bioproducts. Among them, there is growing interest in the group of biopolymers known as polyhydroxyalkanoates (PHAs). They are biopolyesters accumulated intracellularly by a variety of bacteria as a storage material for carbon and energy (Anderson & Dawes, 1990). Due to their biodegradability, thermoplasticity, or biocompatibility, PHAs have potential in agricultural, industrial, and biomedical applications.

Although PHAs possess highly satisfactory properties, their commercial production via bacteria is currently limited because of their high synthesis costs compared with chemically produced polymers. Therefore, there is a growing need to screen potential bacteria which will be able to convert inexpensive carbon sources into valuable PHAs. Many bacteria have been tested and engineered to produce PHAs using low cost industrial and domestic wastes. Due to environmental reasons and the inevitable depletion of fossil fuels, biofuel production has continuously grown in the last years. An increasing demand for biodiesel production worldwide has led to the surplus of crude glycerol as a major byproduct, which can be used in the pharmaceutical or cosmetics industries, however, costly refining process to achieve a high purity is not economically feasible for small biodiesel plants (Da Silva, Mack & Contiero, 2009). Disposal of biodiesel-derived glycerol is associated with a fee. Moreover, it also creates problems with waste management and water pollution (Sharma et al., 2016). Many studies have been conducted to find a new way of using biodiesel-derived glycerol. Crude glycerol has been adopted in green technologies for conversion into valuable products such as bioethanol (Seta et al., 2018), biohydrogen (Haron et al., 2018), 1,3-propanediol (Hiremath, Kannabiran & Rangaswamy, 2011). Crude glycerol has been also considered as a feedstock for PHA production making this cost-effective bioprocess more feasible. The PHA synthesis process from glycerol has been observed in several microorganisms. Recently, De Albuquerque et al. (2018) reported that glycerol from the biodiesel industry was efficiently used by *Cupriavidus necator* for PHAs synthesis. Also, the experiments conducted by De Paula et al. (2017) confirmed significant potential for a new isolate (*Pandoraea* genus) to produce a P(3HB-*co*-3HV) copolymer using crude glycerol. However, these residues have not been tested as a substrate for the fermentative synthesis of PHAs by *Aeromonas* bacteria. Generally, there are few reports of PHA production by wild type *Aeromonas* spp. Some strains of *Aeromonas* spp. seem to show advantages compared to other bacteria due to their robust growth and simple growth requirements, which make them attractive targets for potential PHA production (Liu et al., 2011).

Therefore, in this study, the utilization of biodiesel-derived glycerol as the only carbon source for PHAs production by newly isolated *Aeromonas* strains was investigated and compared with pure glycerol. To the best of our knowledge, no effort has been made to produce PHAs from crude glycerol by *Aeromonas* spp.

## Materials and Methods

### Microbial isolation and selection of PHAs producers

Activated sludge samples were collected in spring from an aeration tank at the municipal wastewater treatment plant in Olsztyn (Poland) and placed into 100-ml sterile glass bottles. To collect the samples, the oral permission from Mr. Ireneusz Jankowski, who was the Vice-Chief of the treatment plant, was received. The sludge samples were diluted with sterile saline solution (0.8% NaCl), then a volume of 0.1 ml was spread onto nutrient agar (Oxoid, Hampshire, United Kingdom). The plates were incubated at 30 °C for up to 72 h in the light. Single bacterial colonies were then picked and cultured in lysogeny broth (1% w/v tryptone, 0.5% w/v yeast extract, 1% NaCl) in 15-ml screw-capped tubes at 30 °C with 220 rpm shaking for 24 h.

The isolated strains were selected as potential PHA producers using polymerase chain reaction-based detection of the PHA polymerase gene. One milliliter of each bacterial strain culture was utilized for DNA isolation using the commercial Genomic Mini kit (A&A Biotechnology, Gdynia, Poland) according to the manufacturer’s protocol. The PCR mixture consisted of 50 ng of extracted DNA, 100 μmol of deoxynucleoside triphosphate (Promega, Madison, WI, USA), 1.5 μM MgCl_2_, 0.5 μM of each primer, 1 U of *Taq* DNA polymerase (Invitrogen, Carlsbad, CA, USA), five μl of reaction buffer (500 mM potassium chloride, pH = 8.5; Triton X-100). To detect potential PHA producers, two primer pairs were used (Table 1). The first one, recognizing both short chain length- and medium chain length- PHAs synthase genes was elaborated by Romo et al. (2007) and the second primer pair was specific for both genes responsible for mcl-PHA synthesis (Solaiman, Ashby & Foglia, 2000) (Table 1). PCR was conducted using an Eppendorf® Mastercycler Gradient (Eppendorf, Wesseling-Berzdorf, Germany). The presence of PCR product was confirmed by analysing five μl of the PCR product on a 1.0% agarose gel stained with ethidium bromide.

**Table 1:** Primer pairs used in the study.

Primer	Nucleotide sequence	Target	Reference
*G-D*	5′ GTGCCGCC(GC)(CT) (AG)(GC)ATCAACAAGT 3′	*phaC, phaC1, phaC2*	Romo et al. (2007)
*G1-R*	5′ GTTCCAG(AT)ACAG(GC)A(GT)(AG)TCGAA 3′		
I-179L	5′ACAGATCAACAAGTTCTACATCTT CGAC 3′	*phaC1, phaC2*	Solaiman, Ashby & Foglia (2000)
I-179R	5′GGTGTTGTCGTTGTTCCAGTAGAGGATGTC 3′		
341	5′ CCTACGGGAGGCAGCAG 3′		Muyzer, De Waal & Uitterlinden (1993)
16SR	5′ TACCTTGTTA CGACTTCACCCCA 3′	*16S rRNA*	Rossau et al. (1991)

### Identification of bacteria by 16S rRNA analysis

Genomic DNA was extracted from a single PHA producing colony using the Genomic Mini kit (A&A Biotechnology, Poland) according to the manufacturer’s instructions. Pure bacterial isolates of *Aeromonas* sp. strains were identified by PCR amplification in a mixture containing 50 ng of DNA, 2.5 mM each of deoxynucleoside triphosphate (Promega, Madison, WI, USA), 400 ng of each primer, 10× PCR buffer (500 mM KCl pH 8.5; Triton X-100), 1.5 mM MgCl_2_, 1 U of Taq DNA polymerase (Invitrogen, Carlsbad, CA, USA). The 16S rRNA gene was amplified using the primers described by Muyzer, De Waal & Uitterlinden (1993) and Rossau et al. (1991) (Table 1). PCR was performed in Eppendorf® Mastercycler Gradient (Eppendorf, Germany). PCR products were purified using a Clean-up kit (A&A Biotechnology, Gdynia, Poland) according to the manufacturer’s instruction. The sequence data were compared with those from the GenBank database using the BLAST function available on the National Center for Biotechnology Information database. Sequences were aligned using the ClustalW program (Thompson, Higgins & Gibson, 1994). A phylogenetic tree was constructed by the neighbor-joining method (Saitou & Nei, 1987) implemented by MEGA version 6.0 with the uncorrected p-distance model using nucleotide sequences of the 16S rRNA gene (Tamura et al., 2013). To determine the degree of statistical support for branches in the phylogeny, 1,000 bootstrap replicates of data were analyzed. The 16S rRNA gene sequences were deposited in GenBank under accession numbers: MH270335, MH270336, MH270337 for the *Aeromonas* spp. AC_01, *Aeromonas* spp. AC_02, and *Aeromonas* spp. AC_03, respectively.

### Culture media and carbon sources

Seed cultures were grown in lysogeny broth (1% w/v tryptone, 0.5% w/v yeast extract, 1% NaCl) at 30 °C with shaking (200 rpm) for 16 h before inoculation. Bacteria were cultivated in three different mineral salt media (MSM): (1) non-limited medium (7 g/l KH_2_PO_4_; 3.5 g/l Na_2_HPO_4_·12H_2_O; 5 g/l (NH_4_)_2_SO_4_); (2) nitrogen-limited medium (7 g/l KH_2_PO_4_; 3.5 g/l Na_2_HPO_4_·12H_2_O; 0.5 g/l (NH_4_)_2_SO_4_); (3) phosphorus-limited medium (0.7 g/l KH_2_PO_4_; 0.35 g/l Na_2_HPO_4_·12H_2_O; 5 g/l (NH_4_)_2_SO_4_). Each medium was supplemented with MgSO_4_·7H_2_O (1 g/l) and trace element solution (2.5 ml/l). Each liter of trace element solution contained: 20 g FeCl_3_·6H_2_O, 10 g CaCl_2_·H_2_O, 0.03 g CuSO_4_·5H_2_O, 0.05 g MnCl_2_·4H_2_O, 0.1 g ZnSO_4_·7H_2_O dissolved in 0.5N HCl. All experiments were conducted using crude glycerol from biodiesel industry and pure glycerol (99% purity; Sigma Aldrich, St. Louis, MO, USA) at the concentration of 1% (w/v) (Kachrimanidou et al., 2014; Phukon et al., 2014). Crude glycerol was derived from non-GMO vegetable oil and was kindly provided by Biofuels S.A. Company (Malbork, Poland). This fraction was composed of 85% glycerol, 7% NaCl, 5.5% water, 2% organic matter-of-non-glycerin, and 0.5% methanol.

### Culture conditions for PHA synthesis

The cultivations were carried out in an orbital shaker at 200 rpm, at 30 °C and pH-value adjusted to 7.0. Fermentation shake flasks were inoculated with 5% v/v of the seed and were subsampled for PHA following a one or two step cultivation. One-step PHAs synthesis was conducted in MSM medium for 48 h. In the case of the two-step production, cells were firstly cultivated in lysogeny broth containing glucose (10 g/l) for the first 8 h. Harvested and washed bacterial cells were then transferred into MSM medium supplemented with crude or pure glycerol and incubated for 40 h in the light. All cultivations were conducted in triplicate.

### Analytical procedures and PHA extraction

Cell growth was monitored by measuring the absorbance at 595 nm (OD_595_). Bacterial cells were harvested by centrifugation at 11,200×*g* for 10 min and washed once with ethanol and distilled water. Cell concentration, defined as cell dry weight (CDW) per liter of a culture medium, was determined by weighing lyophilized cells. Lyophilization was achieved by Lyovac GT2 System (SRK Systemtechnik GmbH, Riedstadt, Germany). PHAs were extracted by shaking lyophilized cells in chloroform (purity ≥99.8%; Sigma-Aldrich, St. Louis, MO, USA) for 5 h. The mixture was filtered through No. 1 Whatman filter paper to remove the cellular debris. After mixture filtration, the isolated biopolyesters were precipitated with cold methanol (purity ≥99.8%; Sigma-Aldrich, St. Louis, MO, USA) and then allowed to evaporate at the room temperature.

### Gas chromatography analysis

To evaluate the PHAs composition, the lyophilized cells were suspended in acidified methanol containing 3% v/v H_2_SO_4_ and an equal volume of chloroform (purity ≥99.8%, Sigma-Aldrich, St. Louis, MO, USA). They were then incubated in the oven (Memmert Type SM 400, Memmert, Schwabach, Germany) for 20 h at 100 °C for esterification. The obtained methyl esters were quantified according to the method described by Furrer et al. (2007) using gas chromatography (Varian CP-3800; Varian, Santa Clara, CA, USA) equipped with a Varian VF-5 ms capillary column (30 m × 0.25 mm, film thickness 0.25 μm). Known quantities of pure 3-hydroxyacids (Larodan, Solna, Sweden) were used as standards. The flame ionization detector temperature was 270 °C with the injection port temperature of 250 °C. Initial column temperature was set to 80 °C and next raised with a rate of at 10 °C/min to 240 °C. Total time for analysis of one sample amounted to 16 min.

## Results

### Phylogenetic analysis

**Figure 1:**
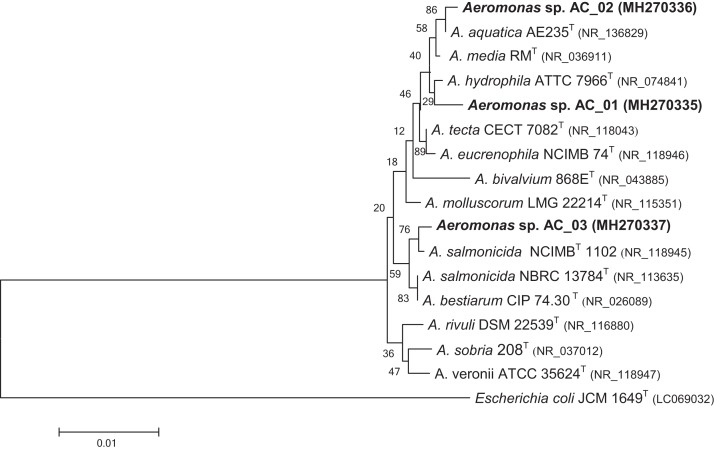
Phylogenetic tree for novel *Aeromonas* sp. AC_01, AC_02, and AC_03 strains. The tree was constructed using neighbor-joining algorithm. *Escherichia coli* JCM 1649 (Acc. no. LC0690032) was selected as the outgroup. Type strains are indicated with superscript T. The numbers on the branches are bootstrap percentages for 1,000 replicates. Characters in parentheses indicate GenBank accession numbers. Bar = 0.01 estimated substitution per sequence position.

The phylogenetic tree demonstrated that the bacterial isolates could be assigned into the *Aeromonas* genus (Fig. 1). The data revealed that isolate AC_01 exhibited the highest similarity with *Aeromonas hydrophila*. Strains AC_02 and AC_03 were determined to be potential members of *A. aquatica* and *A. salmonicida*, respectively.

### Effects of pure and crude glycerol on growth of the isolates

**Figure 2:**
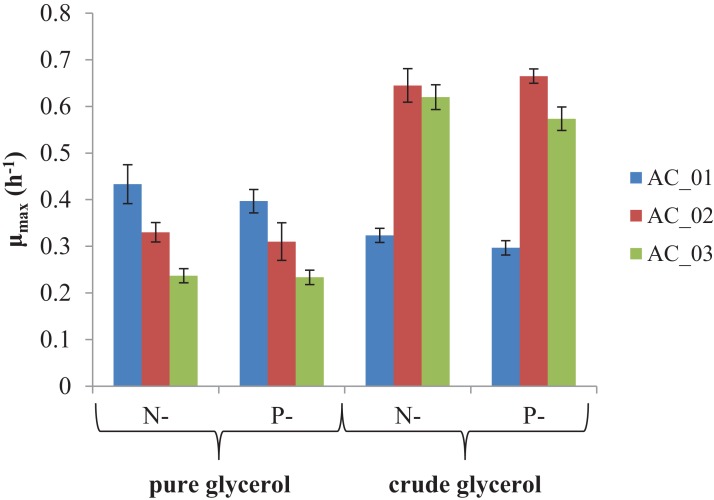
Maximum specific growth rate (μ_max_) of *Aeromonas* sp. strains grown on pure and crude glycerol. Data were expressed as average value and standard deviation of three parallel samples. N, nitrogen-limiting conditions; P, phosphorus-limiting conditions.

The main goal of this study was to evaluate if the by-product from biodiesel production—crude glycerol—could be converted into PHAs. The results showed that all strains were able to utilize the pure and crude glycerol (Fig. 2). During the cultivations, an increase in the absorbance of the culture was observed indicating the bacteria grew. In all experimental treatments, the maximum specific growth rates (μ_max_) were more than 0.2/h. However, two strains, AC_02 and AC_03, reached two and three times higher μ_max_ growing on crude glycerol than on pure glycerol under nitrogen and phosphorus limitation (Fig. 2). The results confirmed that by-product from biodiesel industry did not inhibit the growth of the analyzed *Aeromonas* strains and their growth rate did not depend on applying a macronutrient limitation strategy. CDW of bacteria cultured with crude glycerol was comparable with pure glycerol (Table 2).

**Table 2:** Biosynthesis of PHAs by *Aeromonas* sp. strains grown in shake flasks during one-stage and two-stage process.

Strain	Nutrient limitation	CDW (g/l)	PHA (wt%)	Monomer composition (mol%)	
				3HB	3HV
**(1) One-stage process: ***Cultures were conducted in mineral medium for 48 h*					
***Aeromonas* sp. AC_01**					
Pure glycerol	N	1.69 ± 0.04	7.8 ± 0.09	100	0
	P	1.39 ± 0.01	10.8 ± 0.43	100	0
Crude glycerol	N	2.02 ± 0.11	4.4 ± 0.54	100	0
	P	1.62 ± 0.12	11.1 ± 0.2	100	0
***Aeromonas* sp. AC_02**					
Pure glycerol	N	1.48 ± 0.04	5.0 ± 0.13	89.73	10.27
	P	0.95 ± 0.005	20.7 ± 0.95	100	0
Crude glycerol	N	1.41 ± 0.12	6.5 ± 0.63	100	0
	P	1.22 ± 0.01	10.0 ± 0.21	100	0
***Aeromonas* sp. AC_03**					
Pure glycerol	N	1.20 ± 0.05	3.6 ± 0.09	100	0
	P	0.70 ± 0.005	16.4 ± 0.45	100	0
Crude glycerol	N	1.27 ± 0.03	9.2 ± 0.49	100	0
	P	1.20 ± 0.02	3.3 ± 0.15	100	0
**(2) Two-stage process:** *Cultures were conducted in LB medium for 8 h and then with glycerol for 40 h*					
***Aeromonas* sp. AC_01**					
Pure glycerol	N	2.70 ± 0.12	3.4 ± 0.11	100	0
	P	1.70 ± 0.004	3.0 ± 0.14	100	0
Crude glycerol	N	2.72 ± 0.04	3.6 ± 0.18	100	0
	P	2.33 ± 0.03	3.8 ± 0.14	100	0
***Aeromonas* sp. AC_02**					
Pure glycerol	N	2.88 ± 0.05	3.7 ± 0.16	100	0
	P	3.00 ± 0.05	10.5 ± 0.05	100	0
Crude glycerol	N	2.00 ± 0.10	2.8 ± 0.19	100	0
	P	2.65 ± 0.05	13.6 ± 0.53	100	0
***Aeromonas* sp. AC_03**					
Pure glycerol	N	2.53 ± 0.08	4.7 ± 0.44	100	0
	P	2.32 ± 0.05	42.0 ± 0.57	100	0
Crude glycerol	N	2.58 ± 0.13	5.6 ± 0.40	100	0
	P	3.06 ± 0.09	22.1 ± 0.30	100	0

**Notes:**

Data were expressed as average value and standard deviation of three parallel samples.

3HB, 3-hydroxybutyrate; 3HV, 3-hydroxyvalerate.

In the two-step cultivation procedure, shake-flasks yielded biomass concentrations from 1.7 to 3.06 g/l after 40 h. Lowest biomass was for AC_02 grown on pure glycerol under phosphorus limited conditions and highest biomass was for AC_03 cultivated on crude glycerol under phosphorus deficiency, respectively. As predicted, the CDW value was higher at the end of the two-step cultivations compared with the one-step bioprocess (Table 2).

### PHAs biosynthesis and their chemical characterization

Our results confirmed that the adequate supply of nitrogen and phosphorus during shake-flasks cultures of the analyzed strains did not stimulate the synthesis of PHAs. PHAs were not detected in bacterial cells under non-limiting conditions in the all conducted cultivations. Furthermore, during the two-stage cultivation, lysogeny medium was used as a rich nutrition to support cell growth in the first 8 h. As expected, no PHAs were synthesized during this time. It was observed that nutrient limitation is necessary for the synthesis of significant amounts of PHAs in the analyzed *Aeromonas* strains grown on pure and crude glycerol. Therefore, to estimate the effect of nutrient deficiency on the productivity of PHA synthesis, the one-step and two-step cultivations were conducted under nitrogen and phosphorus limitation. Our findings indicate that using pure and crude glycerol as carbon and energy sources, *Aeromonas* strains were able to synthesize biopolymers in all culture types and feeding regimes (Table 2). However, the PHA content was higher under a phosphorus-limiting environment, except for AC_03 strain grown on crude glycerol during one-stage cultivation, when three times more biopolymers were reached under nitrogen-limiting conditions. The highest PHA concentration was reached during the two-step bioprocess of *Aeromonas* spp. AC_03 cultured on pure glycerol (42% of CDW) under phosphorus limitation. The same strain synthesized two times less PHAs growing on crude glycerol at the same culture conditions and nutrient regime, however it was the highest PHA yield obtained in the cultures supplemented with by-product from biodiesel production.

**Figure 3:**
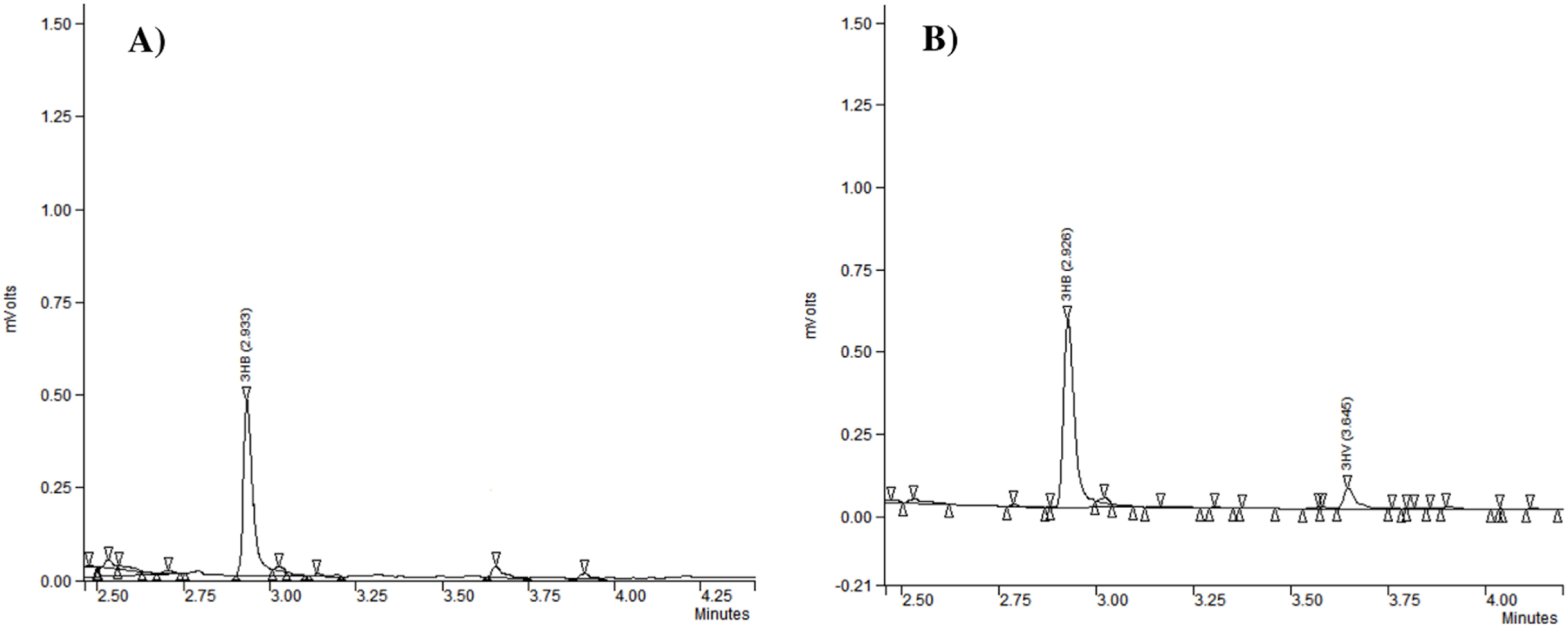
The chromatograms of polyhydroxyalkanoates produced by *Aeromonas* sp. AC_02 on pure and crude glycerol under nitrogen limiting conditions. (A) 3-hydroxybutyrate (3HB); (B) 3-hydroxybutyrate (3HB), and 3-hydroxyvalerate (3HV).

Gas chromatography analysis provided information about the repeat-units composition of the purified biopolymers (Fig. 3). It revealed that generally the PHAs produced by the analyzed strains were polyhydroxybutyrate (P(3HB)) (Table 2). Our study confirmed that besides P(3HB), only *Aeromonas* spp. AC_02 was able to synthesize poly(3-hydroxybutyrate-*co*-3-hydroxyvalerate) copolymer P(3HB-*co*-3HV) under nitrogen limitation in the one-step cultivation supplemented with pure glycerol (Table 2).

## Discussion

The major barrier for industrial applications of PHAs is the high cost of bacterial fermentation. It was suggested that 80% of the final cost of the materials used to produce PHA corresponds to the price of the carbon source (López-Cuellar et al., 2010). Therefore, to make this “bio-based” product more economically reasonable, there is now a global effort to reduce microbial PHA production costs by using cheap waste substrates like biodiesel-derived glycerol (Sharma et al., 2016).

In this study, the new bacteria capable of synthesizing PHAs were isolated from activated sludge in a municipal wastewater treatment plant. These isolates were affiliated to the *Aeromonas* genus and were classified as strains of *A. hydrophila*, *A. aquatica*, and *A. salmonicida* (Fig. 1). Pure commercial glycerol and crude glycerol from a biodiesel production plant were used as the only substrates towards PHA synthesis by the above-mentioned bacteria.

Glycerol, in its pure and waste form, could support cell growth of the analyzed bacterial strains. The biomass concentration of bacterial cells grown on crude glycerol at 48 h was comparable to pure glycerol. This is in contrast with the previous study of Phithakrotchanakoon et al. (2015), which reported three times higher biomass concentration when recombinant *Escherichia coli* was grown on crude glycerol compared with pure glycerol. *C. necator* cultured on waste glycerol reached lower CDW value in comparison to pure glycerol (Cavalheiro et al., 2009). The above mentioned data confirmed that the ability to grow on crude glycerol is dependent on the bacterial species. Furthermore, the microbial growth rate of bacteria cultivated on crude glycerol could be affected by sodium chloride, the main impurity of biodiesel-derived glycerol (Ibrahim & Steinbüchel, 2010). The concentration of NaCl could inhibit bacterial growth and also have a significant effect on PHA productivity and the yield of a final bioproduct (Mothes, Schnorpfeil & Ackermann, 2007). The NaCl content of the crude glycerol used in this study was 0.7 g/l, but did not appear to influence PHA production.

It is well known that various factors could have an impact on the biosynthesis of PHAs, including the bacterium species, growth rate, type of carbon source, culture conditions, or nutrient limitation. Lowering nitrogen and phosphate concentrations should stimulate PHAs synthesis in many bacteria as a response to this stress (Ribeiro, De Souza Silva & Druzian, 2015). Also, the biopolyester content in *Aeromonas* spp. strains seems to be dependent on nutrient limitation (Table 2). Furthermore, our data revealed that the phosphorus deficiency stimulated PHA production by the newly isolated strains better than nitrogen starvation. The production of valuable biopolyesters from crude glycerol has been described for a few species of bacteria (Kumar et al., 2015). Teeka et al. (2012) achieved a high yield of PHAs (41% of CDW) with *Novosphingobium* sp. THA_AlK7 cultivated on biodiesel waste. Shrivastav et al. (2010) reported that *Halomonas hydrothermalis* utilizing 2% Jatropha biodiesel byproduct was able to accumulate 75% of CDW, indicating that in this species crude rather than pure glycerol stimulates better PHA production. The bacterium *C. necator* cultivated on glycerol obtained from the biodiesel industry was able to synthesize PHB in higher concentrations using crude glycerol instead of pure glycerol (De Albuquerque et al., 2018). The same observations were made by De Paula et al. (2017) who employed newly isolated *Pandoraea* sp. MA03, which produced up to 63.6% CDW of PHAs from residual glycerol after 72 h cultivation. In this study, the highest PHA concentration (42% of CDW) was reported in the cultivation supplemented with pure glycerol. Lower yield of PHAs (22% of CDW) was obtained during growth in the medium supplemented with crude glycerol. The negative effect of crude glycerol on PHA productivity could have been due to contamination with sodium chloride. In addition, this value could be enhanced by employing different cultivation strategies. To our knowledge, this is the first reported PHA synthesis by *Aeromonas* spp. using by-product from biodiesel industry.

Several studies have confirmed that bacteria convert pure and crude glycerol into P(3HB) (Kawata & Aiba, 2010; Teeka et al., 2012). Our results confirmed that *Aeromonas* spp. AC_02 is able to incorporate 3HV monomers into the P(3HB) polymer chain. P(3HB-*co*-3HV) copolymer has better properties than PHB, being less fragile and having a lower melting temperature than P(3HB) (Ibrahim & Steinbüchel, 2009). This scl-PHA copolymer seems to be potentially more valuable bioproduct.

## Conclusions

This study showed that pure glycerol and crude glycerol from the biodiesel industry could be converted into short chain length-PHAs by newly isolated strains belonging to *Aeromonas* spp. without the need for refining. Our results clearly demonstrate that these bacteria could be good candidates for additional research on PHAs because of their ability to accumulate high levels of biopolymer. However, further optimization is necessary to improve the bacterial growth rate and biopolymer production efficiency by employing batch-fed approaches and high cell density cultivation methods. Nevertheless, the applied culture conditions and feeding regimes adopted in this study achieved a high P(3HB) content. PHAs from biodiesel-derived byproduct could potentially emerge as the next generation of environmentally-friendly materials with a wide range of applicability.

## Supplemental Information

10.7717/peerj.5838/supp-1 Supplemental Information 1 Raw data.

## Additional Information and Declarations

### Field Study Permissions

The following information was supplied relating to field study approvals (i.e., approving body and any reference numbers): We received oral permission from Mr. Ireneusz Jankowski, who was the Vice-Chief of the wastewater treatment plant in Olsztyn.

### Data Availability

The following information was supplied regarding data availability: GenBank: MH270335, MH270336, MH270337 for the Aeromonas sp. AC_01, Aeromonas sp. AC_02 and Aeromonas sp. AC_03, respectively.
