# Supplementary material for: Exploring nutrient limitation for polyhydroxyalkanoates synthesis by newly isolated strains of Aeromonas sp. using biodiesel-derived glycerol as a substrate
Source: PeerJ. 2018 Oct 23;6:e5838. doi: 10.7717/peerj.5838 (PMC6202957; doi:10.7717/peerj.5838)
Supplement: Supplemental Information 1 [file peerj-06-5838-s001.docx]

**Data for Figure 1**

The 16S rRNA gene sequences are deposited in GenBank under accession numbers: MH270335. MH270336. MH270337

**Data for Figure 2**

***Aeromonas* sp. AC_01**

| Substrate | Nutrient limitation | Maximum specific growth rates (h^-1^) | | | Mean | SD |
| --- | --- | --- | --- | --- | --- | --- |
|  |  | 1^st^ repetition | 2^nd^ repetition | 3^rd^ repetition |  |  |
| glycerol | nitrogen | 0.48 | 0.40 | 0.42 | 0.43 | 0.04 |
|  | phosphorus | 0.40 | 0.37 | 0.42 | 0.39 | 0.02 |
| crude glycerol | nitrogen | 0.34 | 0.32 | 0.31 | 0.32 | 0.02 |
|  | phosphorus | 0.28 | 0.30 | 0.31 | 0.29 | 0.02 |

***Aeromonas* sp. AC_02**

| Substrate | Nutrient limitation | Maximum specific growth rates (h^-1^) | | | | Mean | SD |
| --- | --- | --- | --- | --- | --- | --- | --- |
|  |  | 1^st^ repetition | 2^nd^ repetition | | 3^rd^ repetition |  |  |
| glycerol | nitrogen | 0.32 | | 0.35 | 0.31 | 0.33 | 0.02 |
|  | phosphorus | 0.30 | | 0.27 | 0.35 | 0.31 | 0.04 |
| crude glycerol | nitrogen | 0.63 | | 0.61 | 0.68 | 0.64 | 0.04 |
|  | phosphorus | 0.66 | | 0.68 | 0.65 | 0.66 | 0.02 |

***Aeromonas* sp. AC_03**

| Substrate | Nutrient limitation | Maximum specific growth rates (h^-1^) | | | Mean | SD |
| --- | --- | --- | --- | --- | --- | --- |
|  |  | 1^st^ repetition | 2^nd^ repetition | 3^rd^ repetition |  |  |
| glycerol | nitrogen | 0.24 | 0.22 | 0.25 | 0.24 | 0.02 |
|  | phosphorus | 0.22 | 0.25 | 0.23 | 0.23 | 0.02 |
| crude glycerol | nitrogen | 0.64 | 0.63 | 0.59 | 0.62 | 0.03 |
|  | phosphorus | 0.60 | 0.55 | 0.57 | 0.57 | 0.03 |
